# Do Sociodemographic Factors and Urban Green Space Affect Mental Health Outcomes Among the Urban Elderly Population?

**DOI:** 10.3390/ijerph16050789

**Published:** 2019-03-04

**Authors:** Hyun Jin Lee, Dong Kun Lee

**Affiliations:** 1 Interdisciplinary Program in Landscape Architecture, Seoul National University, Seoul 08826, Korea; hjlee411@snu.ac.kr; 2 Department of Landscape Architecture and Rural System Engineering, Research Institute of Agriculture and Life Sciences, Seoul National University, Seoul 08826, Korea

**Keywords:** aging population, green space, mental health, South Korea, urban health

## Abstract

The mounting mental health issues faced by elderly urban residents increase the social and economic costs to society associated with dementia and depression. Therefore, it is necessary to identify the characteristics of elderly urban residents suffering from mental health issues, to address these issues more effectively. We used 2015 Community Health Survey data from the Korea Centers for Disease Control and Prevention to identify the demographic and social characteristics of 11,408 elderly urban residents in relation to stress levels and symptoms of depression in seven metropolitan areas in Korea, and to calculate the odds ratio for urban green space. We found that the prevalence of these mental health issues generally decreased in relation to the ratio of green space of an area. These findings suggest identifying elderly people who are vulnerable to certain mental health issues based on demographic and social characteristics and demonstrate that the ratio of urban green space within a community is an important component in improving mental health outcomes for elderly urban residents. These findings have policy implications for assisting elderly people vulnerable to certain mental health issues and for establishing a green welfare policy targeting this population.

## 1. Introduction

Increased life expectancy and falling fertility rates are accelerating the aging of populations, and the worldwide population aged over 65 is expected to increase from 524 million in 2010 to nearly 1.5 billion, representing 16% of the world’s population, in 2050 [1]. In Germany, the population aged 65 years or older is expected to increase from 20.7% in 2009 to 29% in 2030 and to 31% in 2050 [1]. However, the fastest increase in the aging population has occurred in East Asia [1]. Korea began to be considered an aging society in 2000, when 7.2% of its total population was aged over 65 years of age; in 2017, this percentage had reached 14.0% (7,257,288 people as at the end of October 2017) and is expected to increase to 24.5% in 2030 and to 28.7% in 2035 [2]. This shifting age structure requires more targeted forms of health and social care and changes in national infrastructures, particularly in relation to healthcare systems [3].

With the development of urbanization in Korea, 91.8% of the population lived in cities in 2017 [4], the percentage of the elderly population living in urban areas rose from 56.4% in 1994 to 76.6% in 2014 [2]. Urban environments often lack access to green spaces due to the proliferation and density of buildings, and urban residents have been found to be more vulnerable to mental health issues such as stress and depression [5,6], due to deterioration in their social and economic status, as well as due to physical illness [1]. Mental health issues among elderly people can also exacerbate dementia [7,8] and increase the suicide rate [9], resulting in an increase in the social and economic costs to society [1,10]. Given that mental health issues occurring among elderly urban residents have implications for society extending beyond the individual level, it is even more necessary to focus on prevention and effective solutions.

Urban green spaces have been shown to provide various health benefits. Recent studies have reported a relationship between mental health and parks and green spaces at the neighborhood level [9,11,12,13,14,15]. Mental health issues may arise because of genetic factors or the psychological state of the individual [16] but can be exacerbated by social and economic inequalities and the state of the surrounding environment [17,18]. Parks and green space can help improve mental health through encouraging physical activity [12,19,20], social interaction, and contact with nature while reducing psychological stress [9,21,22,23]. In addition, it has been reported that people living in environments with greenery, which provides physical, social, and economic benefits to residents, enjoy better mental health than those who do not have access to green space [24].

Previous studies on this topic have mostly used demographic data from people of all ages; thus, little research on the characteristics of elderly households is available. Furthermore, research that is available is limited to national units or specific geographical areas that do not consider environmental characteristics such as urban and rural areas. However, it is important to identify the characteristics of elderly urban households (e.g., single-member household, elderly single-generational household and multi-generational family household) and factors (e.g., health status and health behaviors) affecting mental health to implement effective policies related to elderly urban residents in aging societies. This study thus addresses various gaps in the literature through examining the mental health of the urban elderly and the effects of demographic characteristics and access to urban green space.

The purpose of this study was to investigate the demographic and social characteristics of the elderly who are vulnerable to mental health problems, such as depression and stress, in seven metropolitan areas in Korea. We also investigated the prevalence of mental health problems related to urban green spaces based on the findings of previous studies that urban parks and green spaces are associated with mental health benefits. Furthermore, this study is intended to assist in mental health policy-making and to promote mental health among the growing urban elderly population, and offer practical suggestions concerning welfare policy in relation to green space.

## 2. Materials and Methods

### 2.1. Study Population

We used 2015 Community Health Survey (CHS) data from the Korea Centers for Disease Control and Prevention (KCDC) [25]. The purpose of this survey is to assess the health status, health behavior, and health determinants of Koreans and produce community-based health statistics. The CHS is a survey conducted by trained researchers who visit households sampled nationwide using a multi-stage stratified cluster sampling procedure. The CHS’s target population comprises adults over 19 years of age living in their communities. It is a cross-sectional survey, where participants are sampled each year. CHS data is available to researchers upon request through the KCDC’s online site. Raw data has been provided annually, both at the national and regional levels (one national dataset, and 17 city and province datasets), since 2008. The data are available in text formats. Collected data include sociodemographic information, health behaviors (e.g., smoking, alcohol consumption, physical activity), health status, and subjective health indicators [9,26].

**Figure 1:**
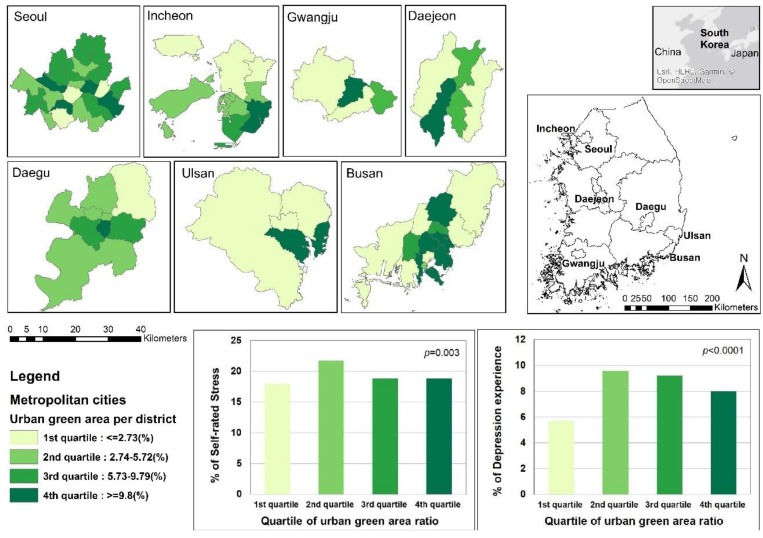
Prevalence of self-rated experiences of stress and/or depression among Koreans aged 65 and over within quartile divisions of urban green areas in seven Korean cities.

We selected seven major Korean metropolitan cities to study the urban elderly population. Detailed geographic information derived from these locations is shown in Figure 1. Among the 11,720 participants aged 65 years and older included from the target areas, questionnaires with missing values among the survey variables were excluded from the analysis. In total, 11,408 people were finally included in the study. This study was approved for exemption by the Institutional Review Board of our university (IRB No. E1902/002-002).

### 2.2. Data

#### 2.2.1. Control Variables

Sociodemographic variables were selected based on previous studies [9,11,14,26], including sex, age, educational level, labor market participation, being a basic livelihood social security recipient, monthly household income, household type, comorbidity, physical activity, cigarette smoking, alcohol consumption, and participation in social activity. The participants were divided according to age groups, as follows: those aged 65–69 years old, those aged 70–79 years old, and those aged 80 years old or older. Educational levels were classified as having completed primary school, middle school or high school, college or university, or having graduated with a Master or higher degree. Participation in the labor market was defined using two answer options (yes, no) to the question “Have you worked in the past week for income purposes?” Participants on temporary vacation were considered labor market participants. Comorbidity was categorized as a disease condition if a participant had been diagnosed by a doctor and was receiving treatment at the time of the survey for two or more diseases. Household type was classified into single-member households, elderly single-generational households (i.e., married couples), and multi-generational family households with children and grandchildren. Physical activity was defined as the number of days in which more than ten minutes of occupational activity or physical activity, such as moderate physical activities (e.g., slow swimming, doubles tennis, volleyball, badminton or table tennis) or athletic activities (e.g., running (jogging), climbing, cycling, rapid swimming or jumping rope) was undertaken during the preceding week. For cigarette smoking, we categorized the participants into current smokers, former smokers and those who had never smoked. In terms of alcohol consumption, participants were classified as alcohol consumers if they had consumed alcohol within the preceding year and non-alcohol consumers otherwise. Participation in social activity was categorized as regular participation in social activity (yes, no) at least once a month.

#### 2.2.2. Response Variables 

The response variables for mental health included subjective stress levels and symptoms of depression. A person’s stress level was classified as high when their answer was “I feel stressed very often or a lot” to the question “How often do you feel stress every day?” However, the answer “I feel stressed a little or rarely” was classified as low. A person was classified as having exhibited symptoms of depression using two response options (yes, no) to the question “Have you ever felt sad or desperate for more than two weeks in the last year?” If the participants answered “yes”, they were classified as having had symptoms of depression.

#### 2.2.3. Explanatory Variables

We used the proportion of urban green area per administrative area derived from CHS data to assess the degree of exposure to green space. Urban green areas include parks and green space (roadsides, road and riverside greenery, small parks, children’s parks, neighborhood parks, theme parks, amusement parks, and green spaces excluding cemeteries), which require little money or time to visit and which are easy to access and use in daily life. Therefore, we selected an existing variable of the ratio of urban green area per administrative area, including park area, to determine the likely amount of exposure to the green environment. We divided them into quartiles with natural breaks to compare the odds ratios of stress levels and symptoms of depression related to the proportion of green areas in various cities (Figure 1). The first quartile refers to the lowest proportion of green area and the fourth quartile refers to the highest proportion of green area per administrative area. In addition, interaction terms between the proportion of green areas and physical and social activities were generated and used as explanatory variables to identify the potential effects of subjective behaviors.

### 2.3. Statistical Analysis

We conducted a frequency analysis and a chi-square test using PASW Statistics 18.0 (SPSS Inc, Chicago, IL, USA) to analyze the demographic and sociological characteristics of the study sample. We conducted a binary logistic regression analysis, with reported symptoms of depression and stress levels as response variables for mental health indicators. We calculated odds ratios according to differences in relation to sociodemographic characteristics and urban green area.

## 3. Results

### 3.1. Study Sample Characteristics

Table 1 shows the sociodemographic characteristics of the study sample by sex. A total of 11,408 people (4922 [43.1%] men and 6486 [56.9%] women) participated in this study, with those aged 65–69 comprising 3865 people (33.9%), those aged 70–79 comprising 5789 (50.7%) people, and those aged 80 or over comprising 1754 (15.4%) people. For household type, 2891 (58.7%) men and 2167 (33.4%) women reported living in elderly single-generational households, and 1608 men (32.7%) and 2587 (39.9%) women reported living in multi-generational family households. Single-member households were more common among women (1732 [26.7%]) than men (423 ([8.6%]). Furthermore, men were more educated than women (*x^2^* = 1736.241, *p <* 0.0001). More women (518 [8.0%]) than men (252 [5.1%]) were social security recipients. Most women (557 [82.6%]) did not participate in the labor market, while a substantial number (143 [35.4%]) of men did. The most commonly reported disease was hypertension (total 558 [50.5%]), and the rate of arthritis was much higher for women (1661 [25.6%]) than for men (338 [6.9%]). Many more men (947 [19.2%]) than women (142 [2.2%]) were cigarette smokers. More men (3245 [65.9%]) than women (2216 [34.2%]) were consumers of alcohol. Most participants did not engage in physical activity (8335 [73.1%]). However, men had a higher rate of physical activity (*x^2^* = 85.0, *p <* 0.0001) and regular social activity (*x^2^* = 74.636, *p <* 0.0001) than women. Finally, 19.3% (2203) of all respondents reported high levels of stress, and 8.0% reported having experienced symptoms of depression.

### 3.2. Association of Sociodemographic Characteristics with Stress Levels and Symptoms of Depression

Table 2 shows the sociodemographic characteristics of the sampled population in relation to stress levels and symptoms of depression. The indicators for having a high risk of stress were: being female, being between the ages of 65 and 69, living in a multi-generational family household, having had a lower level of education, receiving social security payments, having a lower income, comorbidity, participating in the labor market, being a cigarette smoker, engaging in less than three days of physical activity per week, and not participating in regular social activity. The indicators for having symptoms of depression were very similar to the high stress level indicators but had slightly different characteristics. Multi-generational family households reported higher symptoms of depression than single-generational households. All variables except education level and alcohol consumption were statistically significant (*p* < 0.05) for participants who reported high stress levels and symptoms of depression.

### 3.3. Association of the Urban Green Area Ratio with Stress Levels and Symptoms of Depression

Table 3 shows the OR (95% CI) of stress levels and symptoms of depression among the sample population in quartiles according to the urban green area ratio. The fourth quartile was the respective reference category for the response variables. In Models 1 and 2, where the potential variables were adjusted relative to the unadjusted model, the OR for both stress levels and symptoms of depression tended to increase as the ratio of the green area decreased; from the fourth quartile with the highest green area ratio to the first quartile with the lowest green area ratio. After complete adjustment (Model 2a), prevalence of stress levels increased by 2.2% (OR: 1.022, CI: 0.892–1.171) for participants in the third quartile and by 18.3% (OR: 1.183, CI: 1.034–1.353) for participants in the second quartile compared to those in the fourth quartile, the highest green area ratio.

In the case of symptoms of depression, there was a 26.9% (OR: 1.269, CI: 1.056–1.541) increase of participants in the third quartile and a 28.0% (OR: 1.280, CI: 1.047–1.540) increase of participants in the second quartile compared to the fourth quartile. However, both stress levels and symptoms of depression scarcely appeared in the first quartile, the lowest green area ratio. Apart from the first quartile, there was a clear tendency for stress levels and symptoms of depression to increase as the urban green area ratio decreased (*p* < 0.005).

## 4. Discussion

In this study, we first examined the relationship between stress levels and symptoms of depression and the sociodemographic characteristics of elderly urban residents. Elderly women were more likely to experience higher levels of stress and symptoms of depression than elderly men. Some studies suggest that the lower economic status of elderly women compared to elderly men might negatively affect their mental health [23,27]. Most elderly Korean women have been housewives, with no independent income. It has been reported that fewer opportunities for labor market participation can limit the possibility of forming social relationships, which can lead to depression [27]. Those elderly people who do participate in the labor market experienced more stress but were less likely to experience symptoms of depression. These results suggest that encouraging labor market participation as opportunities (e.g., volunteer, join social groups, etc.) for forming social networks among the elderly could be a promising way to reduce the occurrence of depression among this population. However, the stress caused by labor market participation would need to be mitigated through improvements in the working environment for elderly working people. Interestingly, multi-generational family households were found to be more stressful and more linked to symptoms of depression for elderly persons than single-generational households. Within multi-generational families, there is a greater likelihood of differences in political views or economic power among family members, and these differences might cause inter-generational conflict [28,29]. This finding suggests that comprehensive household welfare policies and services are needed to help families live together in multi-generational homes, and to provide support to families where needed to counter the stresses that can occur in such living arrangements.

Current cigarette smokers were more likely to experience higher levels of stress and symptoms of depression. Because this study used a cross-sectional design, these results cannot be interpreted as causal relationships. However, current cigarette smokers may be more likely to experience mental health challenges, as there is a strong association between mental health and personal health behavior, such as smoking [9,26,30,31]. On the other hand, non-consumers of alcohol in our study reported higher rates of stress and symptoms of depression compared to previous studies suggesting that drinking alcohol is associated with poorer mental health outcomes [26,32,33,34]. In Korea, drinking alcohol among elderly people, but not heavy drinking of alcohol, has often been culturally regarded as a social lubricant for communication and alcoholic drinks are served at various social and family functions [35,36]. In this study, it was found that moderate drinking among elderly people had a positive effect on stress relief and in reducing the symptoms of depression. However, the criterion for alcohol consumption in this study did not concern how much was consumed but whether alcohol had been consumed in the preceding year. Although no relationship was found between stress levels and symptoms of depression in relation to physical activity, regular social activity was shown to be positively linked to reducing stress and symptoms of depression [9,26,27,37,38]. Previous studies have reported that social engagement at the neighborhood level could reduce the likelihood of depression caused by air pollution [39] and urbanization [40]. These results suggest the necessity of supporting and increasing opportunities to participate in social activity for the elderly. Overall, the results of this study provide further evidence concerning the importance of identifying relevant sociodemographic characteristics that are likely to affect mental health among elderly urban residents.

In recent years, welfare policies for elderly people have been focused on promoting elderly-friendly environments involving increased opportunities for contact with nature [1,41,42]. Many studies have suggested that there is a positive association between providing green environments in urban areas and mental health benefits [5,43,44,45]. The physical environment affects mental health [44], and environmental improvement at the neighborhood level can contribute to the social integration of elderly people [45,46,47]. Previous studies have assessed the effects on mental health of the density or presence of green space at the neighborhood level, through using the Normalized Difference Vegetation Index (NDVI), a method of measuring the composition of residential greenness [48,49,50]. However, this study was unable to apply these methods to determine relevant environmental indicators because of limited data sources. Therefore, the ratio of urban green area including parks and all open space green areas was used as a quantitative exposure index to determine the extent of the green environment within the urban areas covered in this study.

In this study, no significant relationship was found with stress levels and symptoms of depression in the quartile with the smallest urban green area ratio. Nevertheless, we found that the higher the rate of greenery in a city, the less stress and fewer symptoms of depression reported among its elderly residents. This result supports previous cross-sectional design studies reporting that exposure to the green environment in urban areas has positive relation to mental health among elderly people [51,52]. In addition, because of the interaction effects of the green areas and physical and social activities which may be associated with mental health outcomes were not significant, this study could still identify clear mental health benefits according to the extent of exposure to greener environments in cities.

### 4.1. Policy Implications

We assessed factors affecting the mental health of elderly urban residents in relation to stress levels and symptoms of depression, focusing on the effects of specific sociodemographic factors and varying exposure to green areas. Our results provide substantial empirical data on these aspects within Korea, with policy implications given the uneven extent of certain mental health issues among elderly people. The mental health issues identified in this study could be more effectively targeted with better information concerning the relevant individual characteristics involved and through extending the green environment. Therefore, welfare policies should be implemented for vulnerable groups in terms of mental health, based on relevant data and a commitment to establishing or extending green space to ensure a wider exposure. However, it is not easy to increase the amount of greenery within urban areas in a short period. As an alternative, we propose the development of nature-based activities that utilize urban parks or gardens in order to expose elderly people to green environments in their daily lives. For example, forest bathing (therapy) program is one of the nature-based activities that promote physical and mental health using various elements of forest environment such as landscape, sound, phytoncide and anion [53]. The mental health of elderly people is more related to social activity than physical activity, based on the results of this study. Forest bathing programs provide elderly people with an opportunity to casually form social groups in nature [53] and help them facilitate connectedness with self, neighbors and nature [54]. In addition, resting and engaging in nature reduce fatigue and stress [55] and social contact within nature in parks or gardens boosts health and well-being [56,57]. As levels of association increase within a group, people have been reported to become more open with one another [58]. As a result, positive psychological changes can begin to occur through mutual understanding and interests when social interactions are formed in a green environment [37]. Therefore, for green welfare policies that focus on vulnerable individuals, utilizing urban green spaces is likely to assist in reducing depression and stress for elderly people who lack the opportunities for physical activity and social interaction [53], and also help to reduce the financial burden of healthcare on governments. However, it should be noted that such a suggestion is beyond the scope of this study.

### 4.2. Limitations

This study has some limitations. First, many studies on the relationship between exposure to greenery and health have assessed the level of greenery using NDVI through setting neighborhood units linked to participants’ addresses. In this study, we obtained a green exposure level for the participants using the administrative level of a district (“Gu” in Korean), but addresses could not be obtained. Therefore, it was not possible to precisely define green exposure in terms of individual neighborhood units. In addition, the random effects that could occur at various green levels were not controlled because we reflected the green areas of the administrative districts to the participants. However, the reliability of this study was enhanced through the large sample size used and the quality of the official administrative data collected and released annually. Second, the quality of the green spaces involved was not investigated. However, considering the seasonal characteristics of plants in Korea, and that the CHS is usually carried out from August to October, outcomes in relation to the mental health issues focused on in this study were deemed unlikely to be affected by the seasons and state of the greenery, because the quality of greenery undergoes little change during the survey period. Third, the sample was limited to the residents of seven metropolitan cities. Although populations in all cities were not included, the reliability of the sample was increased through selecting the cities with the largest populations. Fourth, the amount of time participants spent in their residential areas was not considered. If the participants had spent more time in other areas, this could have led to misclassification, due to diverse exposure levels to a green environment with differing durations of exposure. Fifth, one question was used to assess depression among the response variables for mental health outcomes, and only two response options (yes, no) were used. In addition, it is possible that more biased response results were obtained because of the subjective nature of the responses, rather than what might have been obtained using medical diagnoses.

## 5. Conclusions

This study investigated certain sociodemographic characteristics of elderly urban residents, such as their socioeconomic status and health behavior, and the effect of exposure to urban green area on mental health in relation to stress and symptoms of depression. We have identified the characteristics of urban elderly people who are vulnerable to mental health issues and found that the proportion of green areas within a community is an important component in improving their mental health outcomes. Therefore, to ensure ongoing improvements in mental health and maintain the mental health of elderly urban residents, it is critically important to give more attention to identifying vulnerable elderly groups and to either construct new urban green spaces or develop suitable nature-based activities that utilize existing resources.

## Figures and Tables

**Table 1:** Descriptive characteristics of the study sample: 2015 Korean community health survey (*n* = 11,408).

Variable	Category	All		Sex				*x^2^* (*p*)
				Male		Female		
		*n*	%	*n*	%	*n*	%	
**Total**		11,408	100	4922	43.1	6486	56.9	
Age group (years)	65–69	3865	33.9	1758	35.7	2107	32.5	50.857 (0.000)
	70–79	5789	50.7	2540	51.6	3249	50.1	
	80+	1754	15.4	624	12.7	1130	17.4	
Household type	Solitary	2155	18.9	423	8.6	1732	26.7	930.290 (0.000)
	1 generation	5058	44.3	2891	58.7	2167	33.4	
	2, 3 generations	4195	36.8	1608	32.7	2587	39.9	
Education level	Primary school or less	5635	49.4	1377	28.0	4258	65.6	1736.141 (0.000)
	Middle school or high school	4467	39.2	2565	52.1	1902	29.3	
	College or university	1088	9.5	787	16.0	301	4.6	
	Master’s degree or higher	218	1.9	193	3.9	25	0.4	
Financial aid	No	10,638	93.3	4670	94.9	5968	92.0	36.534 (0.000)
	Yes	770	6.7	252	5.1	518	8.0	
Monthly household income in thousands of Korean Won (KRW)	<1000 (<887 USD)	4758	41.7	1746	35.5	3012	46.4	146.626 (0.000)
	1000–3000 (887–2663 USD)	4266	37.4	2093	42.5	2173	33.5	
	3000+ (2663 USD+)	2384	20.9	1083	22.0	1301	20.1	
Labor market participation	No	8536	74.8	3179	64.6	5357	82.6	481.626 (0.000)
	Yes	2872	25.2	1743	35.4	1129	17.4	
Comorbidity prevalence	Hypertension	5758	50.5	2334	47.4	3424	52.8	32.292 (0.000)
	Diabetes	2398	21	1060	21.5	1338	20.6	1.386 (0.239)
	Dyslipidemia	2195	19.2	694	14.1	1501	23.1	147.245 (0.000)
	Arthritis	1999	17.5	338	6.9	1661	25.6	680.139 (0.000)
Cigarette Smoker	Current	1089	9.5	947	19.2	142	2.2	6142.901 (0.000)
	Former	2985	26.2	2795	56.8	190	2.9	
	Never smoked	7334	64.3	1180	24.0	6154	94.9	
Alcohol consumer	No	5947	52.1	1677	34.1	4270	65.8	1131.331 (0.000)
	Yes	5461	47.9	3245	65.9	2216	34.2	
Moderate physical activity (day/week)	0	8335	73.1	3410	69.3	4925	75.9	85.0 (0.000)
	1–3	1407	12.3	628	12.8	779	12.0	
	4+	1666	14.6	884	18.0	782	12.1	
Regular social activity (monthly)	No	5130	45	1986	40.3	3144	48.5	74.636 (0.000)
	Yes	6278	55	2936	59.7	3342	51.5	
Self-rated stress level	Slightly or rarely	9205	80.7	4186	85.0	5019	77.4	105.506 (0.000)
	Very much or a lot	2203	19.3	736	15.0	1467	22.6	
Symptoms of depression		916	8.0	271	5.5	645	9.9	74.656 (0.000)

**Table 2:** Odds ratios (95% Confidence Interval (CI)) of self-rated stress levels and symptoms of depression in relation to sociodemographic characteristics: 2015 Korean community health survey (*n* = 11,408).

Variable	Category	Odds Ratio (95% CI)	
		Self-Rated Stress Level	Symptoms of Depression
Sex	Female	1.564 (1.330–1.839) **	1.577 (1.242–2.003) **
	Male	Reference	Reference
Age group (years)	65–69	1.556 (1.323–1.832) **	1.232 (0.989–1.534)
	70–79	1.419 (1.225–1.642) **	1.068 (0.880–1.296)
	80+	Reference	Reference
Household type	Solitary	0.632 (0.543–0.736) **	1.075 (0.878–1.316)
	1 generation	0.778 (0.690–0.876) **	0.722 (0.603–0.865) **
	2, 3 generations	Reference	Reference
Education level	Primary school or less	1.089 (0.706–1.681)	0.766 (0.404–1.454)
	Middle school or high school	0.944 (0.614–1.451)	0.790 (0.419–1.492)
	College or university	1.022 (0.650-1.607)	0.819 (0.418–1.605)
	Master’s degree or higher	Reference	Reference
Financial aid	No	0.529 (0.446–0.626) **	0.534 (0.433–0.659) **
	Yes	Reference	Reference
Monthly household income in thousands of Korean Won (KRW)	<1000 (<887 USD)	1.707 (1.451–2.008) **	2.086 (1.617–2.690) **
	1000–3000 (887–2663 USD)	1.337 (1.155–1.548) **	1.552 (1.225–1.965) **
	3000+ (2663 USD+)	Reference	Reference
Comorbidity prevalence	No	0.743 (0.672–0.822) **	0.835 (0.723–0.965) **
	Yes	Reference	Reference
Labor market participation	No	0.874 (0.777–0.982) *	1.384 (1.144–1.675) **
	Yes	Reference	Reference
Cigarette Smoker	Current	1.578 (1.304–1.910) **	1.520 (1.153–2.003) *
	Former	0.951 (0.804–1.125)	1.064(1.064–0.831)
	Never smoked	Reference	Reference
Alcohol consumer	No	1.092 (0.984–1.211)	1.226 (1.052–1.429) *
	Yes	Reference	Reference
Moderate physical activity (day/week)	0	1.058 (0.915–1.223)	1.237 (0.983–1.557)
	1–3	1.055 (0.872–1.278)	1.309 (0.976–1.755)
	4+	Reference	Reference
Regular social activity (monthly)	No	1.551 (1.401–1.717) **	1.455 (1.255–1.688) **
	Yes	Reference	Reference

* *p* < 0.05; ** *p* < 0.001.

**Table 3:** Odds ratios (95% CI) of self-rated stress levels and symptoms of depression in quartiles of urban green area ratio.

	Unadjusted Model	Adjusted		
		Model 1	Model 2 (a)	Model 2 (b)
**Self-Rated Stress Levels (*n* = 11,408)**				
1st quartile (≤2.73%)	0.944 ** (0.825–1.081)	0.945 * (0.823–1.085)	0.931 ** (0.811–1.070)	1.163 (0.750–1.804)
2nd quartile (2.74–5.72%)	1.192 ** (1.046–1.359)	1.188 * (1.039–1.358)	1.183 ** (1.034–1.353)	1.451 (0.946–2.227)
3rd quartile (5.73–9.79%)	0.998 ** (0.874–1.140)	1.026 * (0.896–1.175)	1.022 ** (0.892–1.171)	1.492 (0.987–2.256)
4th quartile (≥9.8%)	Reference	Reference	Reference	Reference
*p*-value for trend	<0.005	<0.01	<0.005	0.191
1st quartile (≤2.73%)	0.730 ** (0.590–0.902)	0.740 *** (0.597–0.918)	0.727 *** (0.586–0.902)	0.742 (0.369–1.491)
2nd quartile (2.74–5.72%)	1.276 ** (1.057–1.542)	1.279 *** (1.055–1.550)	1.280 *** (1.047–1.540)	0.820 (0.425–1.583)
3rd quartile (5.73–9.79%)	1.218 ** (1.009–1.471)	1.274 *** (1.052–1.543)	1.269 *** (1.056–1.541)	1.131 (0.615–2.081)
4th quartile (≥9.8%)	Reference	Reference	Reference	Reference
*p*-value for trend	<0.005	<0.0001	<0.0001	0.561

Model 1: adjusted for demographic factors (i.e., sex, age, household type, education, monthly income, financial aid, labor market participation, and comorbidity prevalence); Model 2 (a): adjusted for model 1 + individual behavioral factors (i.e., cigarette smoking, alcohol consumption, moderate physical activity, and regular social activity); Model 2 (b): adjusted for model 2 (a) + interaction of the urban green area ratio with physical activity and regular social activity; * *p* < 0.01, ** *p* < 0.005, *** *p* < 0.001.
